# Clinical Outcomes of Combined Uvulopalatopharyngoplasty and All-Suture Anchor Tongue Base Suspension for Obstructive Sleep Apnea

**DOI:** 10.3390/medicina62101827

**Published:** 2026-09-22

**Authors:** Tzu-Ang Chen, Tsung-Wei Huang

**Affiliations:** 1 Department of Otolaryngology, Far Eastern Memorial Hospital, New Taipei City 220, Taiwan; pasteurchen@gmail.com; 2 Department of Electrical Engineering, College of Electrical and Communication Engineering, Yuan Ze University, Taoyuan City 320, Taiwan

**Keywords:** obstructive sleep apnea, tongue base surgery, tongue base suspension, multilevel airway surgery, all-suture anchor fixation

## Abstract

*Background and Objectives*: All-suture anchor tongue base suspension (TBS) is a recently introduced approach that provides mandibular fixation without a permanent metallic anchor body. Clinical outcome data for this technique when incorporated into multilevel surgery remain limited. This study evaluated clinical outcomes and postoperative complications following multilevel surgery incorporating all-suture anchor TBS in adults with moderate-to-severe obstructive sleep apnea (OSA). *Materials and Methods*: This retrospective single-arm observational study included 30 of 40 consecutive adults with polysomnography-confirmed moderate-to-severe OSA who were intolerant of or declined continuous positive airway pressure therapy and had complete postoperative polysomnography and at least 6 months of follow-up. All patients underwent uvulopalatopharyngoplasty combined with all-suture anchor TBS. Preoperative and 6-month postoperative assessments included the apnea-hypopnea index (AHI), nadir oxygen saturation, Epworth Sleepiness Scale (ESS), snoring visual analog scale (VAS), and body mass index (BMI). *Results*: Median AHI decreased from 50.5 (Q1–Q3, 38.0–68.0) to 15.7 (Q1–Q3, 11.1–21.4) events/h (*p* < 0.001). ESS improved from 16.3 ± 2.5 to 7.5 ± 2.4 (*p* < 0.01), and nadir oxygen saturation increased from 68.5% ± 6.5% to 82.8% ± 9.2% (*p* < 0.01). Surgical success was achieved in 22 patients (73%). Complications were predominantly mild and transient; no infection, suture rupture, or permanent functional deficit occurred. *Conclusions*: Multilevel surgery incorporating all-suture anchor TBS was associated with significant improvements in objective and subjective sleep outcomes at 6 months, with predominantly mild and transient complications. These findings support the feasibility of incorporating this technique into a multilevel surgical protocol; comparative studies are needed to determine the specific contribution of TBS.

## 1. Introduction

Obstructive sleep apnea (OSA) is a common chronic sleep-related breathing disorder characterized by recurrent upper-airway collapse, intermittent hypoxemia, sleep fragmentation, and increased respiratory effort during sleep. Beyond snoring and daytime sleepiness, untreated OSA is associated with cardiovascular disease, neurocognitive impairment, reduced quality of life, and increased health-care burden [1,2,3]. Population-based modeling has estimated that approximately 936 million adults aged 30–69 years worldwide have mild-to-severe OSA and that more than 400 million have moderate-to-severe disease, underscoring the need for durable and acceptable treatment options [4]. Continuous positive airway pressure (CPAP) remains the first-line therapy because of its high physiologic efficacy; however, long-term effectiveness may be limited by intolerance, inadequate nightly use, or refusal of treatment [5,6]. Contemporary American Academy of Sleep Medicine guidance therefore supports discussion of referral for surgical consultation in appropriately selected adults who are unable or unwilling to use positive airway pressure [7].

Upper-airway obstruction in OSA is frequently multilevel rather than confined to a single anatomic segment. Retropalatal collapse is common, but persistent retrolingual narrowing related to posterior displacement or collapse of the tongue base can contribute substantially to residual obstruction in patients with moderate-to-severe disease [8,9]. Palatal surgery alone primarily addresses the retropalatal component and may therefore be insufficient when clinically relevant tongue-base obstruction coexists. This anatomic rationale has led to the development of multilevel surgical strategies combining palatal and hypopharyngeal procedures. Systematic reviews have shown meaningful improvements in objective and patient-reported outcomes after appropriately selected multilevel surgery [8,10], and the sleep apnea multilevel surgery (SAMS) randomized clinical trial further demonstrated that combined palatal and tongue surgery can reduce apnea-hypopnea index (AHI) and daytime sleepiness compared with ongoing medical management in selected patients with moderate-to-severe OSA who have not achieved satisfactory treatment with conventional therapy [11].

Tongue base suspension (TBS) is a tissue-preserving hypopharyngeal procedure intended to stabilize the tongue in a more anterior position through fixation to the mandible, thereby enlarging and supporting the retrolingual airway [12]. Early systems used a soft-tissue-to-bone anchor or bone screw to create an anterior mandibular fixation point, whereas subsequent modifications sought to simplify the operative pathway and reduce implant-related morbidity [12,13,14]. Clinical series combining TBS with uvulopalatopharyngoplasty (UPPP) have reported substantial reductions in respiratory event indices and improvements in symptoms, although outcomes vary with patient selection, surgical technique, and definitions of success [15,16]. A transsubmental approach has also been used in patients with persistent severe OSA after previous palatal surgery, supporting the concept that anterior tongue-base stabilization can be incorporated into different multilevel treatment pathways [17]. Nevertheless, conventional screw-based systems require dedicated instrumentation and leave a rigid implant in the mandible, while some modified suture-based techniques require broader submental or intraoral dissection [13,14,18].

All-suture anchors were originally developed as soft-tissue-to-bone fixation devices for orthopedic applications. Unlike conventional rigid or metallic anchors, they establish osseous fixation through a small cortical tunnel using a predominantly suture-based construct, thereby avoiding a permanent metallic anchor body. Their small footprint and limited requirement for bone removal have supported their use in orthopedic soft-tissue fixation [19,20,21,22].

More recently, we introduced an all-suture anchor-based TBS technique using a Y-Knot^®^ all-suture anchor and monofilament polypropylene suture through a limited submental approach [23]. In this technique, the all-suture anchor design was adapted to create an anterior mandibular fixation point for tongue base suspension. However, biomechanical findings from orthopedic models cannot be directly extrapolated to mandibular TBS because of differences in anatomy and loading conditions. The previous report focused on the operative concept and technical feasibility of the procedure. Cohort-level clinical data describing objective sleep outcomes, patient-reported outcomes, surgical response, and postoperative complications remain limited. The present study therefore evaluated a consecutive series of adults with moderate-to-severe OSA who underwent UPPP combined with all-suture anchor TBS, with the aim of providing a more complete clinical assessment of this technique when used as part of a standardized multilevel surgical protocol.

## 2. Materials and Methods

### 2.1. Study Design and Patient Selection

This retrospective single-arm observational study included consecutive adult patients with moderate-to-severe OSA who underwent multilevel upper-airway surgery at Far Eastern Memorial Hospital between June 2024 and June 2025. The report was prepared with reference to the Strengthening the Reporting of Observational Studies in Epidemiology (STROBE) recommendations for observational studies [24]. The study protocol was approved by the Institutional Review Board of Far Eastern Memorial Hospital (approval number: 115225-E). Eligible patients had polysomnography-confirmed moderate-to-severe OSA, intolerance or refusal of CPAP therapy, and clinical evidence of both retropalatal and retrolingual obstruction based on preoperative assessment and flexible nasopharyngoscopy with the Müller maneuver. Patients with neuromuscular disorders or previous extensive tongue-base surgery were excluded. For the outcome analysis, complete postoperative polysomnography (PSG) and at least 6 months of follow-up were required. During the study period, 40 patients underwent the combined procedure; 10 lacked complete postoperative PSG or adequate follow-up and were therefore excluded, leaving 30 patients in the analytic cohort. Because this was a retrospective assessment of a newly introduced surgical technique, no formal a priori sample-size calculation was performed; all consecutive patients meeting the predefined analytic criteria during the study period were included.

### 2.2. Preoperative Assessment

Preoperative evaluation was designed to characterize OSA severity, symptom burden, body habitus, and the anatomic levels of upper-airway obstruction. Baseline data included age, sex, body mass index (BMI), Epworth Sleepiness Scale (ESS), bed-partner-reported snoring visual analog scale (VAS), overnight PSG, and flexible nasopharyngoscopic assessment. The Müller maneuver was used during awake flexible endoscopy as part of the clinical assessment of dynamic retropalatal and retrolingual narrowing. Friedman tongue position was routinely recorded as part of the preoperative assessment. Drug-induced sleep endoscopy (DISE) was not routinely performed during the study period. In selected patients, a tongue retaining device was used as an adjunctive functional assessment [25]. Only patients in whom both retropalatal and retrolingual obstruction were identified were considered for the standardized combined UPPP and TBS protocol. The decision to proceed with surgery was made after review of OSA severity, anatomic findings, and inability or unwillingness to continue CPAP. Variables used in the present analysis were obtained from the clinical record and sleep-study reports. Baseline AHI and nadir oxygen saturation were taken from the preoperative PSG closest to the operative date.

### 2.3. Surgical Technique

All procedures were performed under general anesthesia according to a consistent operative strategy. UPPP was first performed to address the retropalatal component of obstruction, followed by TBS to provide anterior stabilization of the tongue base. The all-suture anchor procedure was based on our previously described technique [23]. Through a limited submental incision, the anterior mandible was exposed sufficiently to permit anchor insertion. A small tunnel was predrilled in the mandibular cortex, and a Y-Knot^®^ all-suture anchor (CONMED Corporation, Largo, FL, USA) was deployed to establish an anterior mandibular fixation point (Figure 1). The purpose of this construct was to achieve bone-based fixation while avoiding a permanent metallic anchor body.

Before anchor deployment, the manufacturer-supplied braided suture was replaced with a nonabsorbable monofilament polypropylene suture. The polypropylene suture was then passed through the tongue-base musculature and returned anteriorly to the submental fixation site, where it was secured to suspend the tongue base. A fixed numerical depth of suture passage was not used because tongue thickness varies among patients. Passage was maintained within the tongue-base musculature while avoiding excessively deep or lateral trajectories. The lingual point was used as a transoral anatomical landmark to estimate the course of the lingual artery and to guide suture passage while minimizing the risk of vascular injury [26]. The suture was secured under firm but submaximal manual tension sufficient to achieve anterior tongue-base stabilization without visible tissue blanching or focal suture cut-through. Monofilament polypropylene was selected to minimize the amount of braided material retained along a pathway that communicates with the oral tongue-base tissues. The same anchor type, suture concept, operative sequence, and combined palatal/tongue-base strategy were used throughout the cohort to reduce technical heterogeneity. The detailed step-by-step operative concept has been reported previously [23]; Figure 1 illustrates the relationship between the mandibular fixation point and tongue-base suture pathway.

### 2.4. Subjective Evaluation

Daytime sleepiness was assessed with the ESS, an eight-item questionnaire scored from 0 to 24, with higher scores indicating greater subjective sleepiness [27]. Snoring severity was assessed by the patient’s bed partner using a 10-point VAS, ranging from 0 (no snoring) to 10 (maximal snoring) [8]. These subjective measures were recorded preoperatively and again at the 6-month postoperative assessment to complement the objective PSG outcomes.

### 2.5. Objective Evaluation

Overnight PSG was performed preoperatively and at approximately 6 months after surgery. Respiratory events and sleep parameters were scored according to American Academy of Sleep Medicine criteria [28]. Apnea was defined as a reduction in airflow of at least 90% from the pre-event baseline lasting at least 10 s, whereas hypopnea was defined as a reduction in airflow of at least 30% lasting at least 10 s and accompanied by either an oxygen desaturation of at least 3% from the pre-event baseline or an arousal. Because these PSG examinations were performed as routine clinical assessments rather than research-specific evaluations, the sleep technicians were not specifically blinded to the patients’ surgical status. The principal PSG variable was the AHI, expressed as events per hour of sleep. Nadir oxygen saturation was also extracted to characterize the severity of sleep-related hypoxemia. BMI was recorded at the preoperative and 6-month postoperative time points so that changes in sleep outcomes could be interpreted in the context of any interval weight change.

### 2.6. Postoperative Follow-Up and Complication Assessment

Clinical follow-up records were reviewed through at least 6 months after surgery. Postoperative evaluation included direct inspection of the tongue, sublingual region, and floor of the mouth. Flexible laryngoscopy was performed selectively when symptoms or clinical findings indicated the need for further airway evaluation. Postoperative safety assessment focused on complications relevant to tongue-base suspension and the submental approach, including tongue swelling, dysphagia, pain persisting beyond 2 weeks, infection, clinically recognized suture rupture, and permanent functional deficits. Events were recorded from routine postoperative documentation and were categorized according to their clinical course. Transient symptoms that resolved without permanent functional impairment were distinguished from persistent or device-related complications. The 6-month visit served as the principal outcome time point because it allowed postoperative edema and early healing effects to subside before repeat PSG and symptom assessment.

### 2.7. Outcomes and Statistical Analysis

The primary outcome was the change in AHI from baseline to 6 months. Secondary outcomes included changes in ESS, snoring VAS, nadir oxygen saturation, and BMI, together with categorical surgical response and postoperative complications. Surgical success was defined using the commonly applied criterion of a ≥50% reduction in AHI together with a postoperative AHI < 20 events/h [10]. Partial response was defined as a ≥50% reduction in AHI with a postoperative AHI ≥ 20 events/h, and failure was defined as an AHI reduction of <50% [10]. This classification was selected before analysis to permit comparison with previously published OSA surgical series.

Continuous variables are presented as mean ± standard deviation (SD) when distributional assumptions were appropriate or as median with the first and third quartile (Q1–Q3) for nonnormally distributed variables. Normality of paired preoperative-to-postoperative differences was assessed using the Shapiro–Wilk test. Normally distributed paired variables were compared using paired-sample *t* tests, whereas nonnormally distributed paired variables were analyzed using the Wilcoxon signed-rank test. Categorical outcomes are presented as counts and percentages. The surgical success proportion is additionally reported with a 95% Wilson confidence interval to convey the precision of the estimate in this modest-sized cohort. All tests were two-sided, and *p* < 0.05 was considered statistically significant. No imputation was performed for any study variable. Patients without complete postoperative PSG data or adequate follow-up were excluded from the analytic cohort. Statistical analyses were performed using SPSS software, version 19 (IBM Corp., Armonk, NY, USA).

## 3. Results

### 3.1. Study Cohort and Baseline Characteristics

During the study period, 40 patients underwent multilevel surgery incorporating all-suture anchor TBS. Ten patients were excluded from the outcome analysis because of incomplete postoperative PSG or insufficient follow-up, leaving 30 patients with paired clinical data for analysis. To assess potential attrition bias, baseline characteristics were compared between the 30 patients included in the analytic cohort and the 10 patients excluded because of incomplete postoperative PSG or inadequate follow-up. No statistically significant differences were identified in the available baseline characteristics, including age, sex, BMI, preoperative AHI, ESS score, and Friedman tongue position (Supplementary Table S1). Nevertheless, given the small number of excluded patients, non-random attrition cannot be completely excluded. Baseline demographic and clinical characteristics are summarized in Table 1. The mean age was 45 ± 6.7 years, 20 patients (67%) were male, and the mean preoperative BMI was 27.2 ± 3.5 kg/m^2^. Baseline PSG confirmed moderate-to-severe OSA, with a median AHI of 50.5 events/h (Q1–Q3, 38.0–68.0), and the mean baseline ESS score was 16.3 ± 2.5, indicating a substantial burden of daytime sleepiness in the cohort. Regarding upper-airway anatomy, 3 patients had Friedman tongue position grade II and 27 had grade III.

### 3.2. Polysomnographic and Patient-Reported Outcomes

Changes in objective and subjective outcomes are summarized in Table 2. Shapiro–Wilk testing indicated that the paired preoperative-to-postoperative differences in AHI were non-normally distributed, whereas the paired differences in ESS, nadir oxygen saturation, snoring VAS, and BMI were consistent with normality. Accordingly, AHI was analyzed using the Wilcoxon signed-rank test, while the remaining continuous outcomes were analyzed using paired-sample *t* tests. At the 6-month postoperative assessment, median AHI decreased from 50.5 (Q1–Q3, 38.0–68.0) to 15.7 (Q1–Q3, 11.1–21.4) events/h (*p* < 0.001, Wilcoxon signed-rank test). Individual paired changes are shown in Figure 2, which demonstrates the distribution of preoperative and postoperative AHI values across the cohort. Mean ESS improved from 16.3 ± 2.5 to 7.5 ± 2.4 (*p* < 0.01), indicating a parallel reduction in subjective daytime sleepiness. Nadir oxygen saturation increased from 68.5% ± 6.5% to 82.8% ± 9.2% (*p* < 0.01), while snoring VAS decreased from 8.3 ± 1.2 to 4.3 ± 1.4 (*p* < 0.01). BMI remained stable over the same interval (27.2 ± 3.5 vs. 27.4 ± 3.1 kg/m^2^; *p* = 0.41), indicating that the observed changes in sleep-related outcomes occurred without a significant change in body weight.

### 3.3. Surgical Response and Postoperative Safety

Using the predefined response criteria, surgical success was achieved in 22 of 30 patients (73%; 95% confidence interval, 56–86%). Four patients (13%) met criteria for a partial response, and four (13%) were classified as failures. Postoperative complications were predominantly mild and transient. Tongue swelling occurred in five patients (17%). No clinically recognized sublingual hematoma, deep floor-of-mouth edema, or airway compromise was documented. Persistent pain beyond 2 weeks occurred in one patient (3%), and transient dysphagia occurred in one patient (3%). No postoperative infection, clinically recognized suture rupture, or permanent functional deficit was observed during the available follow-up period. Thus, the observed adverse events were primarily early postoperative symptoms rather than persistent implant- or function-related complications.

## 4. Discussion

The present study provides cohort-level clinical outcome data for a recently introduced all-suture anchor TBS technique used within a standardized multilevel surgical protocol. At 6 months, the combined procedure was associated with marked improvements in AHI, nadir oxygen saturation, ESS, and snoring, while BMI remained unchanged. Nearly three-quarters of patients met the predefined surgical-success criterion, and the recorded complications were predominantly transient. These findings extend our earlier technical report [23] by moving from procedural feasibility to a broader assessment of objective sleep outcomes, patient-reported outcomes, response categories, and perioperative safety. At the same time, because every patient underwent both UPPP and TBS, the results should be interpreted as outcomes of the multilevel protocol rather than as proof of the independent efficacy of the tongue-base component.

The magnitude of improvement observed in this cohort is broadly consistent with earlier literature supporting multilevel surgery in selected patients with moderate-to-severe OSA. Previous TBS series have reported meaningful reductions in respiratory-event indices when tongue suspension was combined with UPPP, although reported success rates vary because of differences in baseline severity, anatomy, devices, follow-up intervals, and definitions of response [13,15,16]. In a separate transsubmental series of patients with persistent severe OSA after failed UPPP, tongue-base suspension also produced substantial AHI improvement, illustrating that anterior tongue stabilization may contribute clinically in patients with persistent retrolingual obstruction [17]. More broadly, the SAMS randomized trial showed that combined palatal and tongue surgery can improve AHI and daytime sleepiness compared with ongoing medical management in selected patients with moderate-to-severe OSA [11]. These prior studies support the rationale for a multilevel approach, but they also emphasize that outcomes from combined procedures cannot be attributed to a single surgical component without an appropriate comparison group.

The fixation method is a central technical issue in TBS. Traditional bone-anchor or screw-based systems create a firm mandibular counterpoint for anterior tongue traction but introduce a permanent rigid component and require device-specific instrumentation [12,29,30]. Modified suture techniques reduce dependence on proprietary hardware but may involve larger dissections or different paths of soft-tissue fixation [13,18]. In the present technique, the all-suture anchor was used to preserve the principle of bone-based anterior fixation while avoiding a permanent rigid anchor body. Although orthopedic studies have demonstrated favorable biomechanical performance of all-suture anchors [20,21], these findings cannot be directly transferred to mandibular TBS. All-suture anchor fixation is influenced by cortical structure and thickness [22], while the mandibular application is exposed to different loading directions and repetitive forces generated by tongue movement, swallowing, and speech. Dedicated biomechanical evaluation of the modified mandibular construct is therefore warranted.

A second technical consideration is the choice of suture material along the tongue-base pathway. In the present procedure, the manufacturer-supplied braided suture was replaced with monofilament polypropylene before implantation. Monofilament polypropylene has an established clinical precedent as a suspension suture in previously described tongue base suspension systems. However, replacement of the manufacturer-supplied braided suture represents a modification of the original all-suture anchor construct. Dedicated biomechanical testing of the modified anchor–suture construct was not performed, and equivalence in anchor seating, cyclic displacement, suture–anchor interface behavior, or knot security therefore cannot be assumed. Experimental work has demonstrated greater bacterial adherence to braided than to monofilament sutures in contaminated wound models [31], and a randomized oral-surgery study reported lower microbial adherence and more favorable soft-tissue healing with monofilament polypropylene compared with some multifilament alternatives [32]. These observations provide a rationale for minimizing retained braided material in a clean-contaminated operative field. In the current cohort, no postoperative infections were observed. However, the study did not compare suture materials, and the absence of infection in 30 patients cannot establish a protective effect of polypropylene. Accordingly, this finding should be interpreted descriptively rather than as evidence that monofilament polypropylene reduces postoperative infection risk. Larger comparative studies are required to evaluate whether suture material influences infectious outcomes. Similarly, no clinically recognized suture rupture or fixation failure was observed during the 6-month follow-up; this supports short-term clinical feasibility but should not be interpreted as biomechanical validation of the modified construct. Dedicated biomechanical testing and longer-term follow-up are warranted.

From a practical surgical perspective, an all-suture anchor construct may have several potential applications. Its small osseous footprint may be attractive when the aim is to establish a mandibular fixation point through limited exposure, and the absence of a permanent metallic anchor body may reduce implant bulk. The technique also preserves the familiar principle of anterior tongue-base suspension rather than introducing a fundamentally different mechanism of airway enlargement. For centers already experienced in multilevel OSA surgery, the construct could therefore represent an additional fixation option for selected patients with combined retropalatal and retrolingual obstruction. Nevertheless, the present study did not compare operative time, cost, learning curve, patient comfort, or device-specific quality-of-life outcomes with alternative TBS systems. Those potential advantages remain hypotheses for future comparative evaluation rather than demonstrated benefits of the present series.

The safety findings also require appropriate context. Tongue swelling was the most frequent event and resolved without permanent functional deficit. Persistent pain beyond 2 weeks and transient dysphagia were uncommon, and there were no observed infections, clinically recognized suture ruptures, or permanent deficits. Published device-assisted tongue and hyoid suspension literature has described hardware-related adverse events, including discomfort, extrusion, and infection [14,30]. The absence of these events in the present cohort is encouraging but should be interpreted cautiously because of the limited sample size and 6-month observation period. Late loosening, progressive tissue remodeling, anchor migration, or loss of suspension effect may not be captured by short-term clinical follow-up alone.

The principal limitation of this study is its retrospective, single-center, uncontrolled design. Although no statistically significant differences were identified in the available baseline characteristics between included and excluded patients, non-random attrition cannot be excluded because postoperative treatment response was not available for all excluded patients. Although awake flexible endoscopy with the Müller maneuver was used consistently for preoperative assessment, it does not fully reproduce sleep-state upper-airway collapse. Because DISE was not routinely performed, more detailed characterization of sleep-state collapse patterns was not available in this retrospective study. Future studies incorporating standardized DISE may help refine patient selection for all-suture anchor TBS. In addition, because the PSG examinations were obtained as part of routine clinical care, sleep technicians were not specifically blinded to surgical status, which may represent a potential source of measurement bias. Furthermore, all analyzed patients underwent concomitant UPPP and TBS, so the independent contribution of the all-suture tongue-base procedure cannot be isolated. Follow-up was limited to 6 months, precluding assessment of long-term durability, late complications, or recurrence. The study also did not include serial imaging or direct measurements of tongue-base displacement, anchor position, or fixation mechanics. Accordingly, the absence of clinically recognized fixation failure should not be interpreted as direct evidence of structural stability. Future studies incorporating serial imaging may help evaluate anchor position, tongue-base displacement, and maintenance of suspension over time. These limitations constrain causal inference and generalizability. Strengths include the use of a consistent operative strategy, paired preoperative and postoperative PSG, concurrent patient-reported measures, and explicit reporting of surgical response and complications.

## 5. Conclusions

Combined UPPP and all-suture anchor TBS was associated with significant improvements in polysomnographic and patient-reported outcomes at 6 months, with predominantly mild and transient complications. These findings support the short-term clinical feasibility of this combined multilevel surgical approach. However, because no UPPP-alone comparison group was included, the independent therapeutic contribution of all-suture anchor TBS remains unproven. Prospective controlled studies comparing UPPP alone with UPPP combined with all-suture anchor TBS, together with longer-term follow-up, are needed to determine the specific efficacy and durability of the tongue base component.

## Figures and Tables

**Figure 1:**
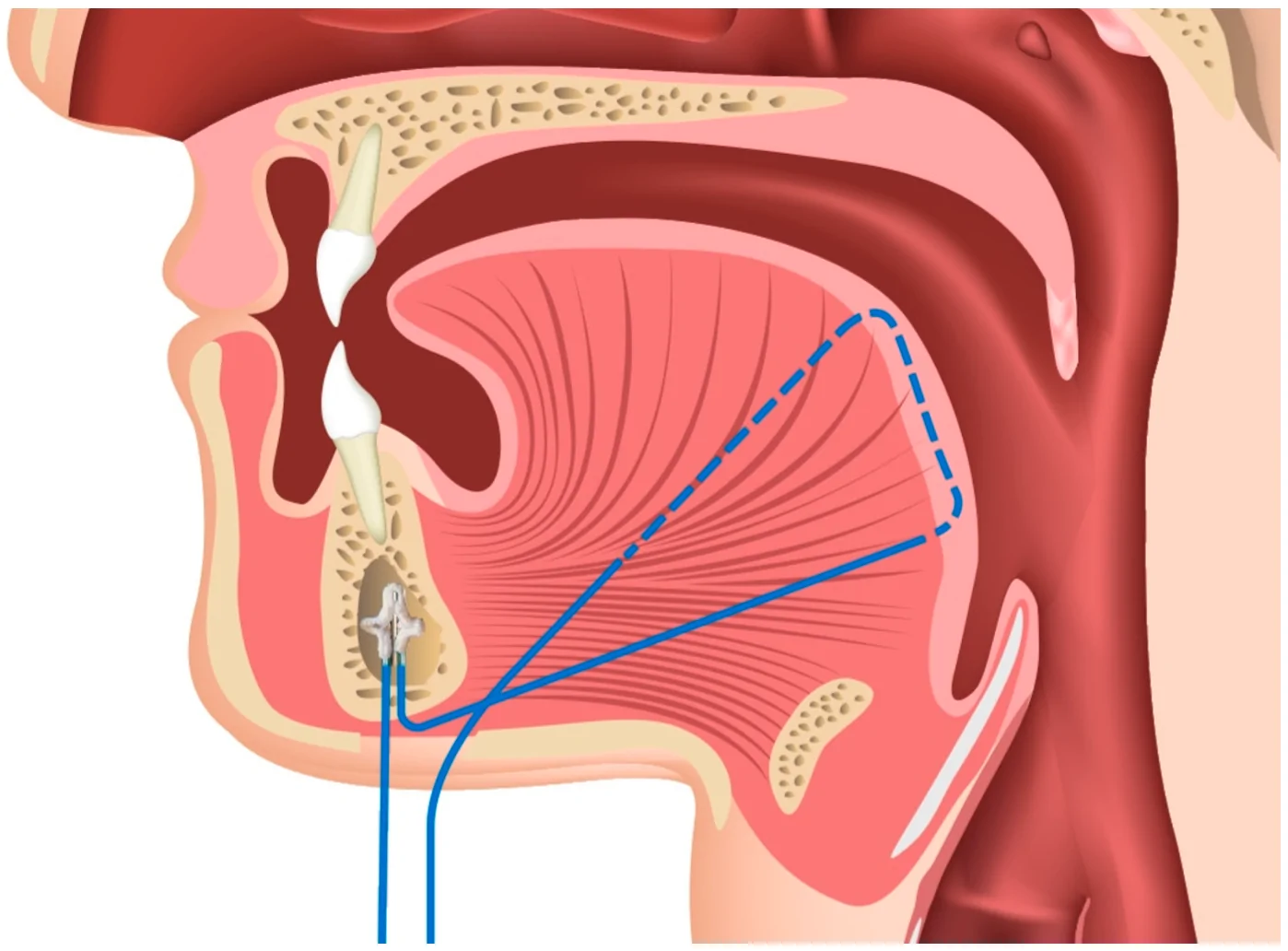
Schematic illustration of all-suture anchor tongue base suspension. The all-suture anchor is deployed within the mandibular cortex, and the monofilament polypropylene suture is passed through the tongue base musculature and secured anteriorly to achieve tongue base suspension.

**Figure 2:**
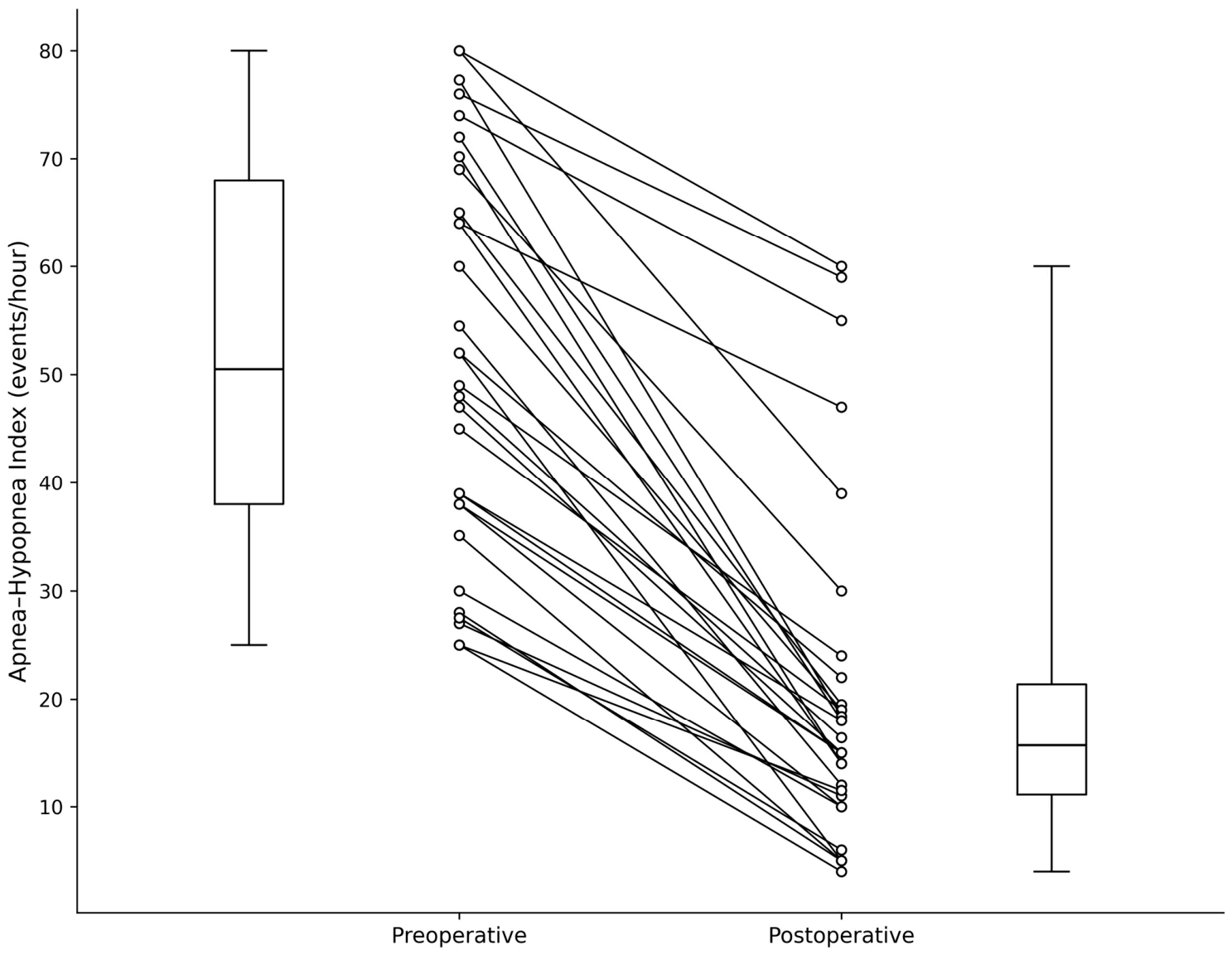
Paired preoperative and postoperative apnea–hypopnea index (AHI) values. Each line represents an individual patient. Boxplots show the median, interquartile range, and full range (minimum to maximum).

**Table 1:** Baseline demographic and clinical characteristics of patients undergoing multilevel surgery incorporating all-suture anchor tongue base suspension.

Variable	Value
Number of patients (*n*)	30
Age (years, mean ± SD)	45 ± 6.7
Sex (Male/Female)	20/10
BMI (kg/m^2^, mean ± SD)	27.2 ± 3.5
Preoperative AHI (events/h)	50.5 (38.0–68.0)
ESS score (mean ± SD)	16.3 ± 2.5
Friedman tongue position (II/III)	3/27

Data are presented as median (Q1–Q3) or mean ± SD, as appropriate. Abbreviations: AHI, apnea–hypopnea index; BMI, body mass index; ESS, Epworth Sleepiness Scale; Q, quartile; SD, standard deviation.

**Table 2:** Comparison of preoperative and postoperative sleep outcomes.

Parameter	Preoperative	Postoperative	*p *Value
AHI (events/h)	50.5 (38.0–68.0)	15.7 (11.1–21.4)	<0.001 ^a^
ESS score	16.3 ± 2.5	7.5 ± 2.4	<0.01 ^b^
Nadir oxygen saturation (%)	68.5 ± 6.5	82.8 ± 9.2	<0.01 ^b^
Snoring VAS	8.3 ± 1.2	4.3 ± 1.4	<0.01 ^b^
BMI (kg/m^2^)	27.2 ± 3.5	27.4 ± 3.1	0.41 ^b^

Data are presented as median (Q1–Q3) or mean ± SD, as appropriate. ^a^ Wilcoxon signed-rank test. ^b^ Paired-sample *t* test. Abbreviations: AHI, apnea–hypopnea index; BMI, body mass index; ESS, Epworth Sleepiness Scale; SD, standard deviation; VAS, visual analog scale.

## Data Availability

The data presented in this study are available from the corresponding author upon reasonable request. The data are not publicly available because of participant privacy considerations.

## Supplementary Materials

The following supporting information can be downloaded at: https://www.mdpi.com/article/10.3390/medicina62101827/s1, Table S1: Comparison of baseline characteristics between patients included in and excluded from the analytic cohort.
