# Supplementary material for: Clinical Outcomes of Combined Uvulopalatopharyngoplasty and All-Suture Anchor Tongue Base Suspension for Obstructive Sleep Apnea
Source: Medicina (Kaunas). 2026 Sep 22;62(10):1827. doi: 10.3390/medicina62101827 (PMC13706835; doi:10.3390/medicina62101827)
Supplement: Supplementary file 1 [file medicina-62-01827-s001.zip › medicina-4574673-supplementary.pdf]

Supplementary Table S1. Comparison of baseline characteristics between patients included in and excluded from the analytic cohort.

Variable	Analytic cohort (n = 30)	Excluded patients (n = 10)	P value
Number of patients (n)	30	10	—
Age (years, mean ± SD)	45 ± 6.7	44.5 ± 7.0	>0.05
Sex (Male/Female)	20/10	7/3	>0.05
BMI (kg/m², mean ± SD)	27.2 ± 3.5	27.5 ± 3.8	>0.05
Preoperative AHI (events/h)	50.5 (38.0–68.0)	47.0 (36.1 – 61.0)	>0.05
ESS score (mean ± SD)	16.3 ± 2.5	16.1 ± 2.7	>0.05
Friedman tongue position (II/III)	3/27	1/9	>0.05

Data are presented as mean ± standard deviation (SD), median (Q1–Q3), or counts, as appropriate. Q1 and Q3 denote the first and third quartiles, respectively. AHI is presented as median (Q1–Q3). Between-group comparisons were performed using Welch's independent-samples t-test for age, BMI, and ESS score, the Mann–Whitney U test for preoperative AHI, and Fisher's exact test for sex and Friedman tongue position. All tests were two-sided.

Abbreviations: AHI, apnea–hypopnea index; BMI, body mass index; ESS, Epworth Sleepiness Scale; SD, standard deviation. The dash indicates that no statistical comparison applies to the group sample sizes.
